# Kidney and urogenital abnormalities in Down syndrome: a meta-analysis

**DOI:** 10.1186/s13052-024-01636-7

**Published:** 2024-04-20

**Authors:** Caterina Maria Rossetti, Giacomo D. Simonetti, Mario G. Bianchetti, Sebastiano A. G. Lava, Giorgio Treglia, Carlo Agostoni, Gregorio P. Milani, J. Peter de Winter

**Affiliations:** 1 Pediatric Institute of Southern Switzerland, Ente Ospedaliero Cantonale, Bellinzona, Switzerland; 2 Faculty of Biomedical Sciences, Università della Svizzera Italiana, via Buffi 13, Lugano, Switzerland; 3 Pediatric Cardiology Unit, Department of Pediatrics, Centre Hospitalier Universitaire Vaudois and University of Lausanne, Lausanne, Switzerland; 4 Heart Failure and Transplantation, Department of Paediatric Cardiology, Great Ormond Street Hospital, London, UK; 5 Academic Education, Research and Innovation Area, General Directorate, Ente Ospedaliero Cantonale, Bellinzona, Switzerland; 6 Pediatric Unit, Fondazione IRCCS Ca’ Granda Ospedale Maggiore Policlinico, Milan, Italy; 7 Department of Clinical Sciences and Community Health, Università degli Studi di Milano, Milan, Italy; 8 Department of Development and Regeneration, Leuven, Belgium; 9 Department of Pediatrics, Spaarne Gasthuis, Haarlem/Hoofddorp, The Netherlands; 10 Leuven Child and Youth Institute, Leuven, Belgium

**Keywords:** Down syndrome, Kidney size, Urogenital abnormalities, Dysfunction of the urinary bladder, Meta-analysis

## Abstract

**Background:**

Reviews on Down syndrome do not or only marginally address the issue of kidney and urogenital tract abnormalities, and lower urinary tract dysfunctions. Hence, we performed a meta-analysis of the literature.

**Methods:**

A literature search was undertaken in the Library of Medicine, Web of Science and Excerpta Medica. The search algorithm combined various keywords: (Down syndrome OR trisomy 21 OR mongolism) AND (kidney OR urinary tract OR bladder) AND (malformation OR dysfunction OR anomaly OR abnormality OR size). The Preferred Reporting Items for Systematic Reviews and Meta-Analyses statement was used.

**Results:**

Eight case-control studies were retained for the final analysis. Three studies addressed the prevalence of kidney and urogenital tract abnormalities: an increased pooled relative risk of 5.49 (95%-CI: 1.78–16.93) was observed in Down syndrome. Penile malformations, obstructive malformations (including urethral valves), dilated urinary tract system, and kidney hypodysplasia were especially common. Three reports addressed the prevalence of lower urinary tract dysfunction: an increased pooled relative risk of 2.95 (95%-CI: 1.15–7.56) was observed. Finally, an autoptic study and an ultrasound study disclosed a reduced kidney size in Down syndrome.

**Conclusions:**

This meta-analysis indicates that abnormalities of the kidney and urogenital tract, lower urinary tract dysfunctions, and a reduced kidney size present with an increased frequency in individuals with Down syndrome.

**Supplementary Information:**

The online version contains supplementary material available at 10.1186/s13052-024-01636-7.

## Introduction

Down syndrome is likely the most recognizable genetic syndrome and occurs in approximately 1 in 600–800 live births without predilection for ethnicity or socioeconomic class. It is a common cause of intellectual disability [1–3]. In addition, people with Down syndrome have a variety of medical conditions and malformations such as somatic growth retardation; hearing loss; obstructive sleep apnea; wheezing airway disorders; otitis media; eye disease, including cataracts and refractive errors; heart defects; gastrointestinal defects; hip dislocation; atlantoaxial instability; autoimmune diseases including hypothyroidism and, less commonly, celiac disease, juvenile idiopathic arthritis, and type 1 diabetes mellitus; and transient myeloproliferative disorder and leukaemia [4, 5].

Available reviews and guidelines on Down syndrome do not or only marginally address the issue of anomalies of the kidney and urinary tract and lower urinary tract dysfunctions [6–10]. Since this matter has never been systematically reviewed, we performed a meta-analysis of the literature addressing these issues.

## Methods

### Search strategy

A literature search was undertaken in the databases of the National Library of Medicine, Web of Science and Excerpta Medica. The search algorithm was a combination of various keywords: (Down syndrome OR trisomy 21 OR mongolism) AND (kidney OR urinary tract OR bladder) AND (malformation OR dysfunction OR anomaly OR abnormality OR size). The literature search was performed in April 2022 in updated in June 2023. Original articles published after 1960 in Dutch, English, French, German, Italian, Portuguese, or Spanish were suitable. We also examined the references of all included articles looking for additional reports. We used the 2020 edition of the Preferred Reporting Items for Systematic reviews and Meta-Analyses statement [11]. After a preliminary selection based on title and abstract, the full text of the selected reports was assessed.

### Eligibility

Orinal reports dealing with kidney and urogenital tract malformations, lower urinary tract dysfunctions [12] and kidney size in Down syndrome were considered eligible. Both prospective and retrospective studies were included. Uncontrolled studies and papers reporting less than 10 subjects with Down syndrome were excluded.

### Data extraction and quality assessment

The following information was extracted from each article using a pilot-tested form: name of the first author, year of publication, country, study design, sample size, and diagnostic methods. Both for cases and controls, data on demographics, reported prevalence of kidney and urogenital tract malformation, lower urinary tract dysfunction, and kidney size were collected. The type of malformation was also extracted. The Newcastle-Ottawa Scale for assessing the quality of non-randomized studies in meta-analyses was used to categorize the quality of the included reports as very low, low, moderate, or high.

Literature selection, data extraction and quality assessment were independently performed by 2 investigators. Disagreements were resolved by consensus. A senior researcher was consulted to solve a controversy concerning 1 paper.

### Data synthesis and analysis

Data synthesis was performed through qualitative and quantitative analysis (meta-analysis). With regard to the meta-analysis, the outcome measures were: 1) the relative risk or risk ratio (RR, main outcome), which is the ratio of the probability of an outcome (kidney and urogenital tract malformations or lower urinary tract dysfunctions) in cases to the probability of the same outcome in controls, 2) the pooled proportion (secondary outcome) of kidney and urogenital tract malformations or lower urinary tract dysfunctions in patients with Down syndrome. Pooled RR and pooled proportions were calculated using a random-effects model. Pooled data are provided with 95% confidence interval values (95%-CI) and displayed using forest plots. Statistical heterogeneity was assessed using the I^2^-index. OpenMeta® software funded by the Agency for Healthcare Research and Quality (Rockville, Maryland, USA) was used.

## Results

### Search results

**Fig. 1:**
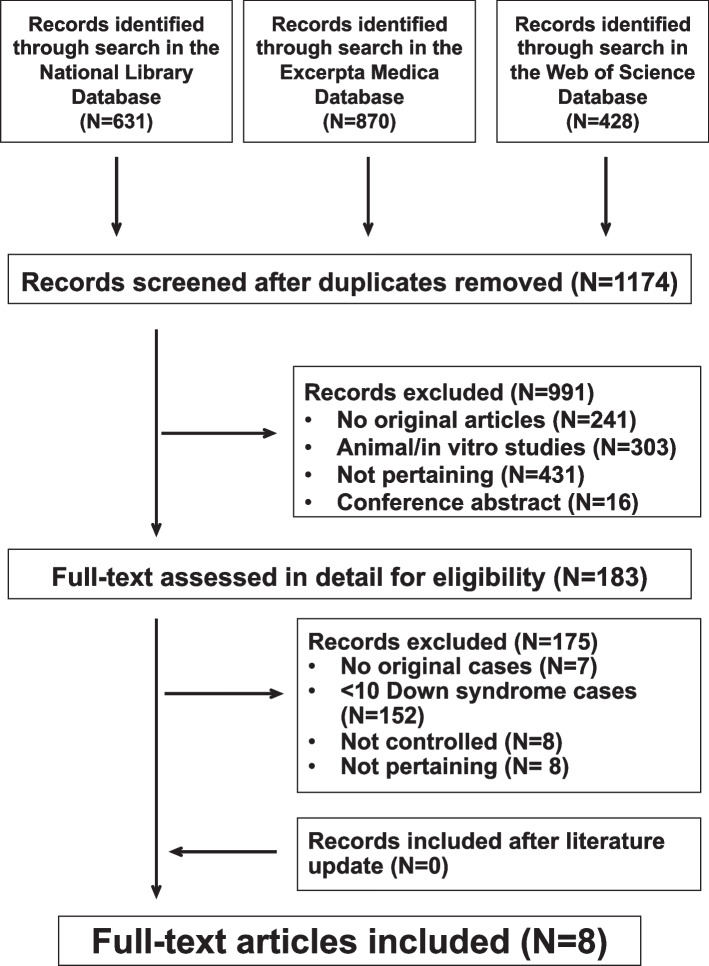
Kidney and urinary tract in children with Down syndrome. Flowchart of the literature search

A total of 8 original papers [13–20] were retained for the final analysis (Fig. 1). They originated from the United States of America (*N* = 5), Belgium (*N* = 1), Japan (*N* = 1) and Turkey (*N* = 1), respectively. The characteristics of the included papers are summarized in supplementary table 1. The study quality rating was high for 1 [14], moderate for 3 [15, 16, 19], and low for the remaining 4 papers [13, 17, 18, 20].

### Anomalies of the kidney and urinary tract

**Fig. 2:**
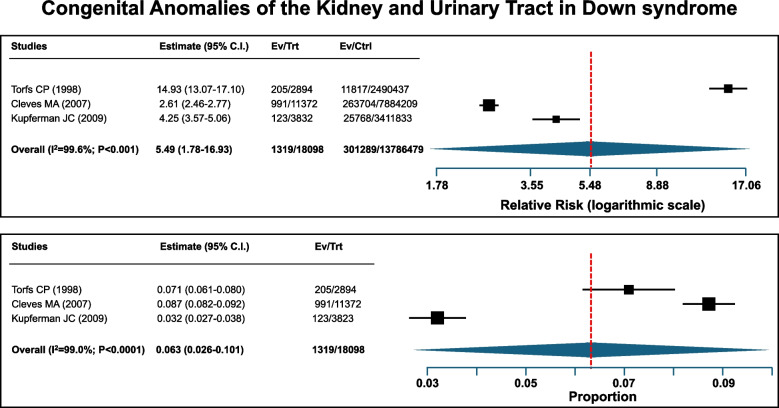
Congenital anomalies of the kidney and urinary tract in Down syndrome. The upper panel depicts the relative risk, i.e. the ratio of the risk in Down syndrome compared to the risk in non affecte subjects. The pooled relative risk was 5.49 (95% CI: 1.78–16-93). The lower panel depicts the proportion of Down patients with a congenital anomaly of the kidney and urinary tract. The pooled proportion was 0.63% (95% CI: 0.26–1.01)

The literature search disclosed 3 case-control studies [14–17], which addressed the prevalence of kidney and urogenital tract abnormalities in a total of approximately 18,000 subjects with Down syndrome and in a control group of more than 13 million subjects. In our review, an increased pooled relative risk of 5.49 was observed in Down syndrome as compared with the control population (Fig. 2, upper panel). A relevant statistical heterogeneity among the 3 studies was also found (I^2^-index 99.6%). The pooled proportion of kidney and urogenital tract malformations among patients with Down syndrome was 0.63% (Fig. 2, lower panel). The following malformations were especially common in Down syndrome (supplementary table 1): penile malformations (hypospadias and epispadias), obstructive malformations (including posterior urethral valves), dilated urinary tract system and kidney hypodysplasia.

An increased prevalence of undescended testes (31 times more frequent) was also detected: 113 out of 18,098 (6.2‰) in Down syndrome as opposed to 2436 out of 13,786,479 (0.2‰) in the general population (*P* < 0.00001).

### Lower urinary tract dysfunctions

**Fig. 3:**
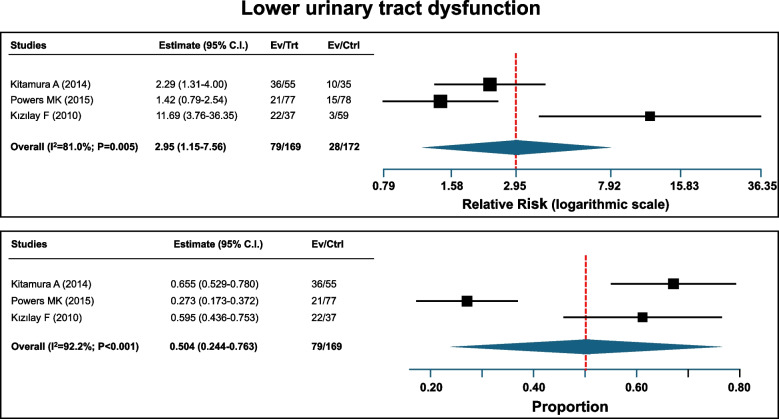
Lower urinary tract dysfunction in Down syndrome. The upper panel depicts the relative risk, i.e. the ratio of the risk in Down syndrome compared to the risk in non affecte subjects. The pooled relative risk was 2.95 (95% C.I.: 1.15–7.56). The lower panel depicts the proportion of Down patients with a lower urinary tract dysfunction. The pooled proportion was 50.4% (95% CI: 24.4–76.3)

Three reports [17–19] including a total of 169 patients with Down syndrome and 172 controls, addressed the prevalence of lower urinary tract dysfunction. An increased pooled relative risk of 2.95 was observed in subjects with Down syndrome (Fig. 3, upper panel). The statistical heterogeneity among the 3 studies was high (I^2^-index 81%). The pooled proportion of lower urinary tract dysfunctions among patients with Down syndrome was 50.4% (Fig. 3, lower panel).

### Kidney size

An autoptic study published in 1967 found that the kidneys’ weight of 21 newborns with Down syndrome was 69% of that in age-matched controls (*P* < 0.01) [13]. A recent ultrasound study disclosed a reduced kidney length and volume in 49 children with Down syndrome as compared to 49 healthy subjects (*P* < 0.001) [20].

## Discussion

The results of the present review point out that individuals with Down syndrome have a five-fold higher risk of kidney and urogenital abnormalities and an almost 3 times higher risk of lower urinary tract dysfunctions as compared to healthy controls. Moreover, the kidney size of individuals with Down syndrome is reduced.

Current guidelines for adults with Down syndrome do not provide any recommendation on kidney and urogenital abnormalities [8]. Pediatric guidelines advise routine postnatal evaluation for kidney anomalies only in cases with prenatal abnormalities [7]. The present meta-analysis detected a 0.6% prevalence of anomalies of the kidney and urinary tract and a 50% prevalence of lower urinary tract dysfunction in Down syndrome. These data support the recommendations available for pediatric subjects. Moreover, these findings point out that a thorough medical history and a careful genital and perineal examination are important in every newborn with Down syndrome. In children, clinicians should routinely look also for symptoms of lower urinary tract dysfunction, such as incontinence, nycturia, increased daytime frequency or weak stream. In case of pathological history or examination, an ultrasound evaluation of the urinary tract and kidney morphology should be performed. Finally, since conventional ultrasound can occasionally fail to detect posterior urethral valves, a voiding cystourethrography deserves consideration in boys with recurrent urinary tract infections or in front of significant postnatal ultrasound abnormalities [21].

Both kidney and urinary tract abnormalities, and lower urinary tract dysfunction may predispose to urinary tract infections [22]. Since this diagnosis is challenging in children with intellectual disabilities, a high index of suspicion is required.

Reduced kidney size was demonstrated in 2 controlled studies. The results of the kidney size studies are supported by uncontrolled investigations, which addressed the creatinine level and the estimated glomerular filtration rate in large Down syndrome cohorts and suggest the need for specific reference values and equations to estimate glomerular filtration rate [23, 24].

In clinical nephrology, the detection of small kidneys suggests the presence of chronic kidney disease. Therefore, the finding of smaller than normal kidneys in people with Down syndrome suggests an increased tendency to develop a chronic kidney disease. An increased prevalence of immune-mediated kidney diseases may also be expected, considering that non-renal autoimmune conditions such as thyroid diseases, celiac disease, juvenile idiopathic arthritis, and type 1 diabetes mellitus are common in Down syndrome [25]. Hence, signs and symptoms consistent with chronic kidney disease should be regularly evaluated, starting from infancy, including measurement of blood pressure and assessment of kidney function. Both kidney replacement therapy [26] and transplantation [27] have been performed in end-stage renal disease patients with Down syndrome.

This study has some limitations. The main is related to the relevant heterogeneity in studies addressing malformations and lower urinary tract dysfunctions. The heterogeneity noted in the studies addressing malformations is likely caused by the different upper age limits of subjects included in the studies [14–17]. The heterogeneity noted in studies addressing lower urinary tract dysfunctions [12] is likely due to the diagnostic criteria, which were based on history alone in 2 studies [18, 19] and more objective criteria in another study [17]. The terminology used to denote both the obstructive urinary tract malformations and the lower urinary tract dysfunctions was inconsistent, and the well-recognized role of sex on the pattern and prevalence of urinary tract malformations and lower urinary tract dysfunctions was partly disregarded in the literature. Furthermore, the quality of some papers was quite poor as well. Finally, the kidney size was evaluated only in 2 studies, thereby preventing a formal meta-analysis of this outcome.

## Conclusions

This meta-analysis demonstrates that a variety of abnormalities of the kidney and urogenital tract, lower urinary tract dysfunctions, and a slightly reduced kidney size are more common in subjects with Down syndrome than in the general population. There is an opportunity to improve the care of children with Down syndrome by providing some specific advice on the management of kidney and urinary tract malformations and dysfunctions [28]. Future efforts should answer the question of whether the increased prevalence of urinary tract malformations translates in an increased occurrence of urinary tract infections and address the long-term nephro-urological outcome at adult age. Finally, a survey to determine the current patterns of evaluation and management of these issues pertaining to kidneys and urogenital tract among Down syndrome Clinics is recommended.

### Supplementary Information

**Additional file 1: Supplementary table 1:** Characteristics of the 8 studies included for the meta-analyses.

## Data Availability

Data and materials are available upon reasonable request to the corresponding author.
