# Supplementary material for: Kidney and urogenital abnormalities in Down syndrome: a meta-analysis
Source: Ital J Pediatr. 2024 Apr 20;50:79. doi: 10.1186/s13052-024-01636-7 (PMC11031854; doi:10.1186/s13052-024-01636-7)
Supplement: Supplementary file 1 — Additional file 1: Supplementary table 1: Characteristics of the 8 studies included for the meta-analyses. [file 13052_2024_1636_MOESM1_ESM.docx]

Supplementary Material

**Supplementary Table 1**: Characteristics of the eight studies included in the meta-analyses.

| **Reference**  **Setting** | **Sample size** | **Study design** | **Relevant outcomes** | **Main findings** | **Limitations** |
| --- | --- | --- | --- | --- | --- |
|  |  |  |  |  |  |
| Torfs (1998)  USA | 2,894 DS and 2,490,437 controls | Retrospective case-control study. Data from the California Birth Defects Monitoring Program; infants until one year of age | Prevalence of:  - obstructive defects of the urinary system  - undescended testes  - hypospadia and epispadia  - renal agenesis  - bladder exstrophy  - absent bladder or urethra  - horseshoe kidney  - bladder duplication | Increase of:  - obstructive defects of the urinary system  - undescended testes  - hypospadia and epispadia  - horseshoe kidney  Similar frequency of unilateral renal agenesis  Not found in DS:  - bilateral renal agenesis  - exstrophy of urinary bladder  - absence of bladder  - urethra or bladder duplication | - Stillbirths and terminations of pregnancy are not included  -DS infants more investigated than controls  -No separate analysis by sex  Obstructive defects not carefully categorized |
| Cleves (2007)  USA | 11,372 DS and 7,884,209 controls | Retrospective case-control study. Data from the Nationwide Inpatient Sample and the Kid’s Inpatient Database; newborns admitted within the first ten days of life | Prevalence of:  - hypospadia and epispadia  - renal agenesis or hypoplasia  - obstructive genitourinary defects  - bladder exstrophy | Increase of:  - obstructive defects  - hypospadia and epispadia  Similar frequency of renal agenesis and hypoplasia  Bladder exstrophy not found in DS | -DS sometimes not confirmed by karyotype  -Stillbirths and terminations of pregnancy are not included  -DS infants are more clinically investigated than controls  -No separate analysis by sex |
| Kupferman  (2009)  USA | 3,832 DS and 3,411,833 controls | Retrospective case-control study. Data from the New York State Congenital Malformation Registry; children up to 2 years of age | Prevalence of renal and urinary tract anomalies | Increase of:  - anterior urethral obstruction  - cystic dysplastic kidney  - hydronephrosis  - hypospadias  - posterior urethral valves  - prune belly syndrome  - renal agenesis  Similar frequency of:  - ectopic kidney  - ureteropelvic junction obstruction  Not found in DS:  - absent ureter  - ureteral atresia or stenosis  - vesicoureteral reflux  - double collecting system  - bladder neck obstruction  - obstruction of urethral meatus | -Information of this registry mainly from inpatient hospital records  - whether stillbirths and terminations of pregnancy included  -Renal hypoplasia, tubular dilation or dysgenesis are not included  -No separate analysis by sex |
|  |  |  |  |  |  |
|  |  |  |  |  |  |
| Kitamura (2014)  Japan | 55 DS and 35 controls | Cross-sectional study. Urological evaluation including physical examination, urinalysis, pre-and post-micturition ultrasound, assessment of bladder volume and post-residual urine, uroflowmetry | Uroflowmetry pattern | DS: pathological uroflowmetry in 66%  Controls: pathological uroflowmetry in 29%  No difference between boys and girls in DS | -Small (<100 subjects) sample size  -No more than one uroflowmetry per case  -Poor quality of uroflowmetry in 9 cases |
| Powers (2015)  USA | 77 DS and 78 controls | Questionnaire developed by the authors | Prevalence of lower urinary tract symptoms | Similar frequency of symptoms in DS (27%) and controls (19%)  No difference in gender for symptoms in ~~DS~~ subjects with DS | -Controls are not age-matched  -Questionnaire not externally validated  -Toilet training status is not considered |
| Kızılay (2020)  Turkey | 37 DS and 59 controls | Prospective cross-sectional study. Overactive Bladder Questionnaire and Dysfunctional Voiding Symptom Score (DVSS); children without a history of a known urological disturbance | Prevalence of dysfunctional voiding | Number of patients with dysfunctional voiding is higher (P<0.001) in DS (59%) than in controls (5.1%) | -Small (<100 subjects) sample size  -No separate analysis by sex  -Incomplete urological assessment |
|  |  |  |  |  |  |
|  |  |  |  |  |  |
| Naeye (1969)  USA | 21 autopsy cases of DS and 26 controls | Cross-sectional autoptic study | Kidney weight | DS kidney weight 69% of controls (P<0.01) | -Small (<100 subjects) sample size  -No karyotype  -Controls not sex-matched |
| Postolache (2022)  Belgium | 49 DS and 49 controls | Prospective cross-sectional study. Doppler and ultrasound of kidney | Kidney length and volume | DS kidney smaller (P<0.001) than controls | -Small (<100 subjects) sample size |
|  |  |  |  |  |  |
